# Reply to Schön et al., "Quality control and considering systematic MIC shifts are key when evaluating the role of* mmpR5 *(*Rv0678*) frameshifts in bedaquiline resistance"

**DOI:** 10.1128/aac.00317-25

**Published:** 2025-08-14

**Authors:** J. Snobre, C. J. Meehan, W. Mulders, L. Rigouts, R. Buyl, B. C. de Jong, A. Van Rie, O. Tzfadia

**Affiliations:** 1 Department of Biomedical Sciences, Institute of Tropical Medicine 37463 , Antwerp, Belgium; 2 Vrije Universiteit Brussel 70493, Brussels, Belgium; 3 Department of Biosciences, Nottingham Trent University 6122, Nottingham, United Kingdom; 4 Faculty of Medicine and Health Sciences, University of Antwerp 81844 , Antwerp, Belgium; City St George's, University of London, London, United Kingdom

## REPLY

We appreciate the opportunity to respond to the comments by Schön et al. (1) and to further discuss the role of frameshift mutations in phenotypic resistance to bedaquiline (BDQ) in *Mycobacterium tuberculosis* (*M. tuberculosis*).

Clinical resistance to BDQ is primarily linked to mutations in *mmpR5*. Mutations in *mmpR5* are spread across the whole gene and observed in isolates with phenotypes ranging from hypersusceptibility to resistance, making interpretation challenging. While the phenotype associated with single-nucleotide variants is usually evaluated using a statistical approach, the WHO endorsed a grading rule that classifies any loss-of-function mutation in *mmpR5* as “associated with resistance—interim.” Accurate classification of frameshift mutations is clinically important as treatment options for patients with BDQ resistance are extremely limited.

In our original article (2), we conducted an analysis of *mmpR5* frameshift mutations listed in the WHO catalogue. We examined the presence of conserved alternative reading frames and late stop codons in 512 isolates, of which 25% were BDQ susceptible. Of 184 unique frameshift mutations with available nucleotide information, 32% contained a late stop codon in the original open reading frame, and 41% preserved a conserved sequence, either in the original or an alternative reading frame. In a smaller data set of 68 isolates with paired phenotypic BDQ MIC data, we investigated whether the presence of late stop codons or alternative reading frames was associated with BDQ susceptibility.

Schön et al. raise concerns about potential batch effects, particularly regarding the data set from Peretokina and Zimenkov (3, 4) for which the MIC values may be systematically low. In these studies, quality control of MIC determinations was performed in each series using the *M. tuberculosis* H37Rv strain (ATCC 27294). Reported MIC values for H37Rv were 0.12 mg/L in 99% of tests using MGIT 960. The MIC distribution for wild-type isolates ranged from ≤0.03 to 1.0 mg/L (mode 0.12 mg/L) in MGIT. When compared with studies included in the WHO 2018 technical report (5), the modes for two studies using MGIT were 0.25 mg/L, which is indeed higher than the mode of 0.12 mg/L reported in the Peretokina data set.

We acknowledge that including data from only four laboratories could have resulted in batch effects in our small data set, especially given the systematically low MICs in the Peretokina study. We also agree with Schön et al. that using non-truncated MICs for H37Rv control strains—rather than testing only at the critical concentration—is a more robust approach for detecting systematic differences.

Drawing on input from Schön et al. and recent evidence, we take this opportunity to revisit three key questions regarding the interpretation of frameshift mutations:

1) Are frameshift mutations always linked to phenotypic resistance in *M. tuberculosis*? Evidence suggests that frameshift mutations do not necessarily result in loss of function and can be compatible with a functional protein and a drug-susceptible phenotype. For *mmpR5*, numerous phenotypically BDQ-susceptible isolates harboring a frameshift mutation have been reported (6–8). Experimentally, a mouse model study showed that BDQ retained bactericidal activity in a strain carrying the *mmpR5*_198insG mutation (9).

A recent study reported frameshift variants distributed widely across the *M. tuberculosis* genome, with isolates carrying frameshift variants in a mean of 112/4,031 genes (10), many being essential genes. Frameshift mutations in the essential *rpoB* gene responsible for rifampicin resistance have been reported (11, 12), and experimental studies have demonstrated that a frameshifted *rpoB* gene can still produce a full-length rpoB protein (13). In essential genes associated with linezolid resistance, 21 of the 22 frameshift mutations reported in the CRyPTIC data set (10) were found in phenotypically susceptible isolates. If frameshift mutations universally caused complete loss of gene function, their presence in essential genes would be incompatible with bacterial survival.

2) What are the biological mechanisms that might allow the gene to retain protein structure and function in the presence of a frameshift mutation? Ribosomal frameshifting, transcriptional slippage, alternative reading frames, and in-frame alternative start codons are mechanisms that may explain how a gene with a frameshift mutation can retain function (13–18). In our study, we hypothesized that this could occur in *M. tuberculosis* through the presence of late stop codons and alternative reading frames. We found that 41% of isolates preserved a conserved sequence, either in the original reading frame, via late stop codons, or through an alternative reading frame. The presence of conserved *mmpR5* sequences despite frameshift mutations observed in our study provides a possible biological explanation for the BDQ susceptibility seen in strains carrying such mutations. Further experimental work is needed to determine to what extent these sequences are translated into functional proteins.

3) Are isolates with frameshift mutations and predicted conserved *mmpR5* sequences associated with BDQ susceptibility? Even if frameshift mutations were experimentally shown to preserve protein structure and function through the hypothesized mechanisms, it should still be demonstrated that this results in a susceptible phenotype. In our study, we observed a correlation between the presence of conserved sequences and BDQ susceptibility in a small set of isolates with paired MIC data. As Schön et al. remarked, batch effects may have biased our results. A larger data set is thus needed to validate this hypothesis.

In conclusion, numerous exception reports of *mmpR5* frameshifts in phenotypically susceptible isolates, along with biological insights from other bacterial systems, challenge the WHO rule classifying all *mmpR5* frameshift mutations as associated with resistance. As highlighted by Shon et al., larger data sets are needed to test whether conserved sequences are a biological mechanism explaining BDQ susceptibility in the presence of a *mmpR5* frameshift mutation. This work will be essential to ensure that BDQ can be included in the treatment for all patients who may benefit from it.
